# Sleep quality and its associated factors among patients with tuberculosis: A cross-sectional study

**DOI:** 10.3389/fpubh.2022.1047425

**Published:** 2023-01-04

**Authors:** Xiangmin Liu, Huizhen Lan, Xinyu Bai, Qian Li, Yan Wen, Mei Feng, Xiangdong Tang

**Affiliations:** ^1^Department of Respiratory and Critical Care Medicine, West China School of Nursing, West China Hospital, Sichuan University, Chengdu, China; ^2^Ward 3, Department of Tuberculosis, The Fourth People Hospital of Nanning, Nanning, China; ^3^Sleep Medicine Center, Mental Health Center, Translational Neuroscience Center, West China Hospital, Chengdu, China

**Keywords:** tuberculosis, sleep disturbance, TB-related stigma, perceived stress, immune, nutrition

## Abstract

**Background:**

Tuberculosis (TB) patients commonly suffer from sleep issues owing to various adverse drug reactions (ADRs), disease symptoms, and the contagious nature of their disease. These sleep issues negatively affect the treatment outcome and quality of life. However, the prevalence of sleep disturbance and its associated factors among TB patients have rarely been reported.

**Methods:**

A total of 497 inpatients with TB from three hospitals in China were enrolled in this cross-sectional study to investigate their sleep quality using the Pittsburgh sleep quality index (PSQI). Clinical data, including demographic information, TB-related stigma, perceived stress, and nutrition- and immunity-related indicators, were also collected to explore the factors associated with sleep disturbance among the recruited patients.

**Results:**

Approximately 70% of the recruited patients reported a sleep disturbance to varying degrees, presenting poorer global and subjective sleep qualities, longer sleep latency, shorter sleep duration, lower sleep efficiency, more frequent sleep disturbances, greater use of sleeping medication, and more severe daytime dysfunction. Furthermore, the body mass index (BMI), hemoglobin levels, albumin levels, and T lymphocyte count of the patients in the poor sleep quality group were significantly lower than those in the good sleep quality group (*p* < 0.05). Increasing age, higher income, drug resistance, higher stigma or stress perception, lower albumin levels, and lower CD4 levels were significantly associated with sleep disturbance among TB patients (*p* < 0.05).

**Conclusion:**

Three-quarters of the participants were found to suffer from a probable sleep disturbance. And sleep problems are linked to biological traits that interact with psychological, cultural, and social factors in complex ways. It is therefore important to pay attention to the sleep quality of TB patients, especially those with the identified risk factors. Besides, taking care of these risk factors may prove to be an effective sleep management strategy.

## Introduction

Tuberculosis (TB) is still considered a pandemic that is caused by *Mycobacterium tuberculosis* (Mtb) (1). With an estimated 10.6 million new cases and a mortality rate of 15% worldwide in 2021, it is the second leading infectious killer after COVID-19 and the 13th leading cause of death worldwide (2). Current TB treatment strategies rely heavily on long-term anti-TB regimens involving multiple medications (3). Among TB patients, various adverse drug reactions (ADRs), such as liver injury and neuropathy, or disease symptoms, such as cough, dyspnea, and pain, often lead to a high prevalence of sleep disturbance (4). TB with the comorbidity of sleep disturbance often puts patients at risk for other chronic diseases, such as diabetes mellitus, hypertension, psychiatric disease, and substance-use disorders (5, 6). Moreover, chronic insomnia is associated with disturbances in immune regulation, manifested by a decrease in the levels of lymphocytes (CD3, CD4, and CD8 cells), which can worsen TB outcomes (7). Therefore, it is crucial to provide immediate attention and timely intervention to TB patients suffering from sleep disturbances.

Sleep quality is determined by their satisfaction with their sleep experience, which comprises the aspects of sleep initiation and sleep maintenance (8). Insomnia and disorders that disturb the quality of sleep account for most sleep disturbances (9). Previous studies have demonstrated sleep quality to be strongly associated with several factors. A recent study reported that stigma, which is marked by labeling, stereotyping, and prejudice, has a strong relationship with self-reported characteristics of sleep deficiency, including insomnia and sleep duration (10), and this correlation was significantly mediated by stress and depression (11). Accumulating evidence also suggests that perceived stress is a significant risk factor for reduced sleep quality (8, 12). In addition, clinical studies have demonstrated that sleep quality exhibits a reciprocal relationship with nutrition and immunity (13, 14).

Similar to acquired immune deficiency syndrome (AIDS) patients, TB patients also suffer from a persistent and deep-rooted stigma, primarily stemming from discrimination and avoidance from others, difficulties in service utilization and employment, and so on. It has been reported that about 42% to 82% TB patients suffered from varying levels of stigma (15). Another study showed that more than half of the TB patients perceived higher stress, which increased the risk of emotional distress (16). Previous studies have shown that the presence of at least one chronic disease, such as diabetes, in TB patients is closely associated with psychological distress (17). The incidence of TB is accompanied by a decrease in appetite, nutrient malabsorption, and metabolism disturbance, leading to wasting and undernutrition status (18). Undernutrition in TB patients manifests as protein-energy malnutrition and micronutrient deficiencies, which contribute to immunodeficiency, decrease in BMI, hypoalbuminemia, anemia, and lymphocytopenia in patients, thus inhibiting their ability to fight infection (19, 20).

The epidemiological reporting of sleep disturbance in TB patients is rarely considered, and there exists a dearth of literature correlated with its predictors, in particular with stigma, perceived stress, nutrition- and immune-related indicators (4, 21). Thus, this work investigated and analyzed sleep quality and its associated factors among TB patients in China.

## Materials and methods

### Study design and settings

This cross-sectional survey was approved by the Biomedical Research Ethics Committee of West China Hospital, Sichuan University. Through convenience sampling, we recruited 497 TB patients who underwent treatment at West China Hospital, Guangyuan Mental Health Center, and the Fourth People′s Hospital of Guangxi in China between November 2021 and February 2022. Clinical data, including demographic information, sleep quality, TB-related stigma, and perceived stress, were collected using appropriate instruments on the day of admission. Fasting venous blood samples were collected the morning after the day of admission for examination of nutrition and immunity markers.

### Inclusion and exclusion criteria

The inclusion criteria were as follows: (1) Confirmed or highly suspected TB patients who have been diagnosed based on the guidelines of the national TB program (17, 22); (2) Patients who could complete the questionnaire with their communication and cognitive skills; and (3) Patients who voluntarily participated in the study.

The exclusion criteria were as follows: (1) Patients with confirmed psychiatry disorders or severe somatic disorders, including but not limited to tumors, critical infections, and immune diseases; and (2) Patients who were administered immune-altering medications or infusion of blood products.

### Measurements

#### Clinical evaluation

Demographic information, including age, gender, height, weight, education years, marital status, income per month, duration of illness, drug resistance status, and whether the patient suffered from diabetes, was collected using an electronic structured questionnaire. Drug resistance was defined as the resistance of Mtb to at least one first-line anti-TB drug, such as isoniazid and rifampicin, in the drug susceptibility test (23).

#### Evaluation of sleep quality, TB-related stigma, and perceived stress

Sleep quality was measured using the Pittsburgh sleep quality index (PSQI) during the last month of the recruitment (8). The questionnaire comprises 19 individual items that generate seven “component” scores: subjective sleep quality, sleep latency, sleep duration, sleep efficiency, sleep disturbances, use of sleeping medication, and daytime dysfunction. Scores for these seven components are added up to obtain a global score ranging from 0 to 21. Higher global scores indicated worse sleep quality. A PSQI global score of >5 was defined as poor sleep quality, while ≤ 5 was defined as good sleep quality (4). Previous research has shown that the Chinese version of the PSQI is highly reliable and appropriate for the Chinese population (24).

TB-Related Stigma Scale (TRSS) was used to assess the stigma of TB patients. This scale has been proven suitable for measuring stigma among Chinese TB patients. It uses a 4-point Likert scale and comprises nine items to provide a total score (range: 0–27). Higher scores indicate greater stigma (25).

The Chinese version of the 14-item Perceived Stress Scale (PSS-14) was used to evaluate the psychological distress during the previous month of the recruitment. It is a 5-point Likert scale and items 4, 5, 6, 7, 9, 10, and 13 were scored in reverse. The total score ranged from 0 to 56 and a higher score indicates more perceived stress (8).

All the self-reported scoring materials employed in this study have been validated and widely used in clinics, exhibiting an excellent Cronbach's alpha of 0.814~0.925.

#### Nutrition and immunity markers

Clinical data, including diagnosis, prescriptions, and laboratory test results, were extracted from the electronic medical record. The nutrition and immunity parameters primarily comprised hemoglobin, albumin, and *T* cell subset (CD3, CD4, and CD8 counts, and the CD4/CD8 ratio).

### Statistical analysis

SPSS (version 21.0) was used for statistical analyses. Continuous variables were presented as means ± standard deviation (SD), and group differences were examined using the independent sample′s *t*-test. Categorical variables were presented as frequencies and percentages, and group comparisons were performed using the Chi square test. Logistic regression analysis was conducted to identify the risk factors related to sleep disturbance. A *p*-value of < 0.05 was considered statistically significant.

## Results

### Prevalence and demographic characteristics

Initially, a total of 512 TB patients were recruited for the study. Of these, 15 patients were eventually excluded owing to illogical or incomplete responses to the questionnaires, with a participation rate of 97.1%. Finally, 497 participants were enrolled with a mean age of 50.9 ± 18.3 years (range: 14–89 years), comprising 282 men (56.5%) and 215 women (43.5%). Based on the Health Industry Standard of the People's Republic of China—Classification for Tuberculosis (WS196–2017), those TB patients were classified as pulmonary, extrapulmonary, or mixed (26). The average PSQI, TRSS, and PSS-14 scores were 8.8 ± 4.6, 8.5 ± 5.7, and 39.1 ± 8.3, respectively. Using a cut-off score of 5 points, 344 (69.2%) patients were reported to be suffering from sleep disturbances. Patients were divided into two groups based on their PSQI global score: poor sleep quality and good sleep quality. Furthermore, according to PSQI self-reported data in the group of poor sleep quality, more than 36% of this population reported daytime dysfunction ≥3 times per week, and approximately 16% used medicine to improve sleep quality.

We began by examining the participants' sleep habits. Patients with poor sleep quality had significantly higher scores for global, subjective sleep quality, sleep latency, sleep duration, sleep efficiency, sleep disturbances, use of sleeping medication, and daytime dysfunction than those with good sleep quality (*p* < 0.05) (Table 1).

**Table 1:** Sleep quality in patients with tuberculosis.

**Item**	**Good sleep quality** **(*N* = 153)**	**Poor sleep quality** **(*N* = 344)**	***t***	***P***
Global PSQI	3.9 ± 2.0	10.8 ± 3.0	−27.887	< 0.001
Subjective sleep quality	0.8 ± 0.5	1.6 ± 07	−13.553	< 0.001
Sleep latency	0.8 ± 0.6	2.0 ± 0.8	−18.004	< 0.001
Sleep duration	0.3 ± 0.4	1.2 ± 0.8	−15.153	< 0.001
Sleep efficiency	0.4 ± 0.6	1.8 ± 0.8	−17.437	< 0.001
Sleep disturbances	0.8 ± 0.5	1.9 ± 0.7	−18.060	< 0.001
Use of medication	0.01 ± 0.08	0.30 ± 0.03	−6.858	< 0.001
Daytime dysfunction	0.9 ± 0.6	2.1 ± 0.8	−17.206	< 0.001
Daytime dysfunction	0.9 ± 0.6	2.1 ± 0.8	−17.206	< 0.001

PSQI, Pittsburgh Sleep Quality Index; P < 0.05.

We then looked at the participants' demographics, disease characteristics, stigma, and perceived stress. We discovered that poor sleepers were older, had fewer education years, and had a higher stigma or perceived stress. Furthermore, TB patients with poor sleep quality had a higher proportion of the population who were divorced or widowed, had drug resistance, or had diabetes than those with good sleep quality (*p* < 0.05). There were no differences in sex or illness duration between the good and poor sleep groups (*p* < 0.05) (Table 2).

**Table 2:** Demographic characteristics, stigma and perceived stress in patients with tuberculosis.

**Item**	**Good sleep quality** **(*N* = 153)**	**Poor sleep quality** **(*N* = 344)**	***t*/*F*/χ^2^**	***P***
Age, years	46.0 ± 18.8	53.1 ± 17.7	−4.012	< 0.001
Male, *n* (%)	81 (52.9%)	201 (58.4%)	1.300	0.254
Education, years	10.2 ± 4.1	9.4 ± 4.1	2.024	0.044
**Marital status**, ***n*** **(%)**				
Unmarried	36 (23.5%)	44 (12.8%)	11.714	0.003
Married	111 (72.5%)	269 (78.2%)		
Divorced or widowed	6 (3.9%)	31 (9.0%)		
**Monthly income**, ***n*** **(%)**				
≤ 3,000	120 (78.4%)	237 (68.9%)	5.259	0.072
3,000–5,000	21 (13.7%)	60 (17.4%)		
>5,000	12 (7.8%)	47 (13.7%)		
**Duration of illness**, ***n*** **(%)**				
≤ 12 months	96 (62.7%)	233 (67.7%)	1.177	0.278
>12 months	57 (37.3%)	111(32.3%)		
Drug resistance, *n* (%)	5 (3.3%)	50 (14.5%)	13.659	0.001
Diabetes, *n* (%)	13 (8.5%)	52 (15.1%)	4.082	0.043
TRSS	5.8 ± 4.8	9.8 ± 0.8	−7.346	< 0.001
PSS-14	34.0 ± 8.0	41.3 ± 7.5	−9.633	< 0.001

TRSS, Tuberculosis-related Stigma Scale; PSS-14, Perceived Stress Scale; P < 0.05.

### The nutrition and immune characteristic

Third, we examined the participants' BMI, hemoglobin, albumin, and T lymphocytes. All of the data in the poor sleep quality group were significantly lower than those in the good sleep quality group (*p* < 0.05) (Table 3).

**Table 3:** Nutrition and immune parameters in patients with tuberculosis.

**Item**	**Good sleep quality** **(*N* = 153)**	**Poor sleep quality** **(*N* = 344)**	**F/H**	***P***
**Nutrition**				
BMI, kg/m^2^	21.0 ± 3.5	20.2 ± 3.3	2.431	0.015
Hemoglobin	120.0 ± 20.6	113.8 ± 24.0	2.681	0.008
Albumin	38.5 ± 5.7	35.2 ± 7.4	4.712	< 0.001
**T lymphocyte counts**				
CD3, cell/ul	918.4 ± 405.8	742.7 ± 439.4	3.454	0.001
CD4, cell/ul	526.0 ± 252.1	430.9 ± 251.5	3.383	0.001
CD8, cell/ul	394.2 ± 231.2	269.3 ± 182.5	2.809	0.006
CD4/CD8	1.6 ± 0.8	1.8 ± 1.0	−2.277	0.024

BMI, body mass index; P < 0.05.

### Logistic regression analysis of factors associated with poor sleep quality in TB patients

According to logistic regression analysis, increasing age, higher income, drug resistance, higher stigma or stress perception, lower albumin levels, or lower CD4 counts all had a significant association with poor sleep quality in TB patients (*p* < 0.05) (Table 4).

**Table 4:** Logistic regression analysis of associated factors in tuberculosis patients with sleep disturbance.

**Item**	**With sleep disturbance**		
	**OR**	**95% CI**	***P***
Age	1.029	1.003–1.055	0.028
Education	0.984	0.880–1.099	0.770
**Marital status**	
Married	1.000	Reference	0.523
Unmarried	2.125	0.579–7.798	0.256
Divorced or widowed	1.013	0.254–4.028	0.986
**Monthly income**	
≤ 3,000	1.000	Reference	0.005
3,000–5,000	2.333	1.245–8.920	0.017
>5,000	3.606	1.487–21.131	0.011
Duration of Illness (≤ 12 months)	0.943	0.460–1.937	0.874
Drug resistance	6.240	1.057–36.848	0.043
Diabetes	1.109	0.421–2.925	0.834
TRSS	1.161	1.077–1.250	< 0.001
PSS-14	1.163	1.095–1.236	< 0.001
**Nutrition**	
BMI, kg/m^2^	0.908	0.820–1.005	0.062
Hemoglobin	0.996	0.979–1.013	0.619
Albumin	0.935	0.882–0.991	0.025
**T Lymphocyte counts**	
CD3	1.000	0.990–1.012	0.052
CD4	0.993	0.980–0.999	0.024
CD8	0.993	0.985–1.001	0.072
Constant	0.023		0.113

TRSS, Tuberculosis-related Stigma Scale; PSS-14, Perceived stress scale; BMI, Body mass index; *P* < 0.05.

## Discussion

We investigated the prevalence of sleep disturbance and its associated factors among TB patients, which had previously been reported only infrequently. In this study, approximately 70% of the patients reported varying degrees of sleep disturbance, with poorer global sleep quality and subjective sleep quality, longer sleep latency, shorter sleep duration, lower sleep efficiency, more frequent sleep disturbances, increased use of sleeping medication, and more severe daytime dysfunction which was higher than a study of 17% in India (4). The differences could be attributed to different measurement tools, as well as the recruitment of a large number of critical patients with complex conditions. Furthermore, no statistically significant differences in PSQI global score were found among patients with different types of tuberculosis (*F* = 1.331, *p* = 0.265).

The current study also discovered that patients who were divorced or widowed, having drug resistance or diabetes, had poor sleep quality (9.0% vs. 3.9%, 14.5% vs. 3.3%, 15.1% vs. 8.5%). Divorced or widowed people were more likely to have sleep problems. One possible explanation is that a lack of companionship does not provide them with the emotional support they require to sleep well (27). Furthermore, diabetes patients were more prone to have sleep disturbances, probably owing to hyperglycemia and altered insulin homeostasis (5, 28). Furthermore, our study found that TB patients with poor sleep quality had worse nutritional status and cell immune function than those with good sleep quality.

In a logistic regression analysis, increasing age, higher income, drug resistance, a higher score of stigma or perceived stress, a lower level of albumin, and lower CD4 *T* cell counts were found to be predictors of poor sleep quality in TB patients. TB was more common in older people, and the notification rate and mortality rate increased with age (29). Our findings are consistent with the current literature, which suggests that the elderly is more vulnerable to sleep disorders. The reason for this could be that as we age, the circadian system and sleep homeostatic mechanisms become less robust as the amount and pattern of sleep-related hormone secretion changes (30). Our study found an increased risk of poor sleep quality with higher income, which contradicted a previous study (27), possibly because this population has experienced more work stress (such as TB-related stigma). Patients with drug resistant tuberculosis (DR-TB) are more susceptible to psychosocial issues or sleep problems than patients with drug susceptible tuberculosis, possibly due to the complexity and prolonged duration of treatment (often 24 months or longer), overwhelmingly higher cost, serious drug toxicity (especially neuropsychiatric toxicity), which seriously affects the curative effect and results in a high mortality rate (31, 32). Furthermore, treatment options for DR-TB are limited, and it is not always possible to discontinue or substitute medications, despite the neuropsychiatric toxicity that causes sleep disturbances (31).

People avoid and isolate groups at risk of TB transmission, despite the fact that their original purpose was to prevent disease (33). Stigma has been identified as a major barrier to good treatment adherence and a driver of TB transmission (15, 34). It can have a negative impact on relationships, psychological well-being, and sleep quality as a mediator and moderator (10). Excessive or prolonged perceived stress can activate the hypothalamic-pituitary-adrenal (HPA) axis, releasing hormones such as cortisol, which can suppress the immune system and lead to sleep deficiency (35, 36), with an underlying biopsychosocial mechanism that includes neurobiological underpinnings such as neuroendocrine changes, as well as psychosocial factors such as a lack of social support. Our study, like Fu's among COVID-19 survivors (33), found that patients with higher perceived stigma or stress had a higher risk of poor sleep quality. These findings suggest that sleep disturbances may be caused by social and psychological factors other than TB disease and ADRs. Finding solutions to sleep problems by reducing TB-related stigma and stress may be especially important for people with TB.

Chronic diseases frequently compromise the body's nutritional and immune systems. Undernutrition has consistently been linked to increased TB incidence and mortality, clinical manifestation severity, and poor treatment outcomes (19, 37). Hypoalbuminemia and lymphocytopenia, in particular, are risk factors for mortality in TB patients (38). Albumin serves several functions and is commonly used in clinical nutritional assessment (39). Previous findings suggest that low albumin levels and sleep disturbances may have a mutual effect. Inflammation is thought to be an upstream factor that affects both sleep-wake disorder and malnutrition in a synergistic manner (40). Another biological mechanism influencing systemic metabolic rate is an increase in physiological indicators of hyperarousal in adults with objectively short sleep durations, which leads to an increase in cortisol and norepinephrine levels (39). Similarly, sleep and immunity may have a directional relationship and interact with one another. Immune system activation influences sleep, and sleep circadian rhythms organize innate and adaptive immune processes and functionalities (13, 41). Mtb infection and chronic stress have been shown to reduce immunity, as evidenced by changes in the number and function of antigen-specific *T*-cells, such as a lower percentage of circulating CD4 *T* cells (42, 43). A significant decrease in CD3, CD4, and CD8 *T* cell counts in TB patients indicates significant immunosuppression or immune deterioration, which is closely related to the severity of TB illness (44). Depending on the magnitude and duration of the inflammatory response elicited by microbial challenges, it can reduce or increase sleep duration and intensity, but also disrupt sleep (13). Elizabeth has also concluded that bacterial infections can affect sleep quality, possibly through the influence of certain immune-inflammatory response components (45). For another, clinical data show that sleep affects various immune parameters such as lymphocyte subpopulation counts and activity (e.g., CD4, CD8) and cytokine levels (e.g., IFN-g, TNF-a) (13, 45, 46). In line with the previous studies, our study found that T lymphocytes were reduced; specifically, lower CD4 *T* cell counts were a key predictor of poor sleep quality in TB patients. The findings suggest that detection of nutritional and immunological status should be strengthened for the management of TB patients with sleep disturbances, who may benefit from improved albumin levels and CD4 *T* cell counts; and ideal sleep quality is fundamental to nutritional status and immune function, both of which are beneficial for TB treatment.

There are some limitations to the current study. First, the study is cross-sectional, which does not lend itself to a causal explanation. Moreover, we used a self-reported sleep questionnaire rather than objective methods, such as polysomnography (PSG) or actigraphy, which allow for more precise measurement; thus, potential inaccuracies in data reporting may exist.

## Conclusion

TB remains a major clinical and public health challenge. The study indicated that TB patients had a high incidence of sleep disturbance, as well as an elevated stigma and perceived stress, poorer nutritional status and cell immune function when compared to those who did not have the symptoms. Sleep problems are linked to biological traits that interact with psychological, cultural, and social factors in complex ways. Patients with TB who have these risk factors require more attention. Besides, taking care of these risk factors, which include psychosocial and biological factors, such as offering counseling and nutritional and immunological support to TB patients, may prove to be an effective sleep management strategy.

## Data availability statement

The data that support the findings of this study are available from the corresponding author upon reasonable request.

## Ethics statement

The studies involving human participants were reviewed and approved by the Biomedical Research Ethics Committee of West China Hospital, Sichuan University. Written informed consent to participate in this study was provided by the participants' legal guardian/next of kin.

## Author contributions

XL and XT designed the study. HL, XB, and QL collected the data. XL analyzed the data and wrote the article. MF and YW provided guidance on study design and methods. XT assisted in this article revision. All authors have read and approved the final manuscript.

## References

[1] Seki M Choi H Kim K Whang J Sung J Mitarai S. Tuberculosis: a persistent unpleasant neighbour of humans. J Infect Public Health. (2021) 14:508–13. 10.1016/j.jiph.2021.01.005 33743373

[2] World Health Organization. Global Tuberculosis Report 2022 Factsheet. (2022). Available online at: https://www.who.int/publications/m/item/global-tuberculosis-report-2022-factsheet (accessed October 26, 2022).

[3] Yu X Li L Xia L Feng X Chen F Cao S. Impact of metformin on the risk and treatment outcomes of tuberculosis in diabetics: a systematic review. BMC Infect Dis. (2019) 19:859. 10.1186/s12879-019-4548-4 31623569 PMC6796338

[4] Raj JP Ramesh N. Quality of sleep among patients diagnosed with tuberculosis-a cross-sectional study. Sleep Breath. (2021) 25:1369–77. 10.1007/s11325-020-02242-7 33169242

[5] Matteson-Rusby SE Pigeon WR Gehrman P Perlis ML. Why treat insomnia? Prim Care Companion J Clin Psychiatry. (2010) 12:PCC.08r00743. 10.4088/PCC.08r00743bro 20582296 PMC2882812

[6] Yuan Y Heizhati M Wang L Li M Lin M Gan L. Poor sleep quality is associated with new-onset hypertension in a diverse young and middle-aged population. Sleep Med. (2021) 88:189–96. 10.1016/j.sleep.2021.10.021 34781033

[7] Savard J Laroche L Simard S Ivers H Morin CM. Chronic insomnia and immune functioning. Psychosom Med. (2003) 65:211–21. 10.1097/01.PSY.0000033126.22740.F3 12651988

[8] Xu H Yang X Lai X Zhao C Tu X Ding N. Longitudinal relationships among perceived stress, suicidal ideation and sleep quality in Chinese undergraduates: a cross-lagged model. J Affect Disord. (2022) 299:45–51. 10.1016/j.jad.2021.11.033 34813870

[9] Bian SZ Zhang L Jin J Zhang JH Li QN Yu J. The onset of sleep disturbances and their associations with anxiety after acute high-altitude exposure at 3700 m. Transl Psychiatry. (2019) 9:175. 10.1038/s41398-019-0510-x 31332159 PMC6646382

[10] Nwanaji-Enwerem U Condon EM Conley S Wang K Iheanacho T Redeker NS. Adapting the health stigma and discrimination framework to understand the association between stigma and sleep deficiency: a systematic review. Sleep Health. (2022) 8:334–45. 10.1016/j.sleh.2022.03.004 35504839 PMC9233012

[11] Wang Z Dang J Zhang X Moore JB Li R. Assessing the relationship between weight stigma, stress, depression, and sleep in Chinese adolescents. Qual Life Res. (2021) 30:229–38. 10.1007/s11136-020-02620-4 32909163

[12] Chung KF Cheung MM. Sleep-wake patterns and sleep disturbance among Hong Kong Chinese adolescents. Sleep. (2008) 31:185–94. 10.1093/sleep/31.2.185 18274265 PMC2225574

[13] Besedovsky L Lange T Haack M. The sleep-immune crosstalk in health and disease. Physiol Rev. (2019) 99:1325–80. 10.1152/physrev.00010.2018 30920354 PMC6689741

[14] Doherty R Madigan S Warrington G Ellis J. Sleep and nutrition interactions: implications for athletes. Nutrients. (2019) 11:822. 10.3390/nu11040822 30979048 PMC6520871

[15] Chen X Du L Wu R Xu J Ji H Zhang Y. Tuberculosis-related stigma and its determinants in Dalian, Northeast China: a cross-sectional study. BMC Public Health. (2021) 21:6. 10.1186/s12889-020-10055-2 33397334 PMC7780403

[16] Mohammedhussein M Hajure M Shifa JE Hassen TA. Perceived stigma among patient with pulmonary tuberculosis at public health facilities in southwest Ethiopia: a cross-sectional study. PLoS ONE. (2020) 15:e0243433. 10.1371/journal.pone.0243433 33290413 PMC7731994

[17] Chen X Wu R Xu J Wang J Gao M Chen Y. Prevalence and associated factors of psychological distress in tuberculosis patients in Northeast China: a cross-sectional study. BMC Infect Dis. (2021) 21:563. 10.1186/s12879-021-06284-4 34118910 PMC8196916

[18] Kant S Gupta H Ahluwalia S. Significance of nutrition in pulmonary tuberculosis. Crit Rev Food Sci Nutr. (2015) 55:955–63. 10.1080/10408398.2012.679500 24915351

[19] Guo X Yang Y Zhang B Cai J Hu Y Ma A. Nutrition and clinical manifestations of pulmonary tuberculosis: a cross-sectional study in Shandong province, China. Asia Pac J Clin Nutr. (2022) 31:41–8. 10.6133/apjcn.202203_31(1).0005 35357102

[20] Dargie B Tesfaye G Worku A. Prevalence and associated factors of undernutrition among adult tuberculosis patients in some selected public health facilities of Addis Ababa, Ethiopia: a cross-sectional study. BMC Nutr. (2016) 2:7. 10.1186/s40795-016-0046-x

[21] Itagi ABH Dipankar SP Krishna Veni D Yunus GY. Evaluation of spirometric measures and quality of sleep in tuberculosis patients and their non-tuberculosis family caregivers. Cureus. (2021) 13:e17788. 10.7759/cureus.17788 34659999 PMC8496126

[22] Bresenham D Kipp AM Medina-Marino A. Quantification and correlates of tuberculosis stigma along the tuberculosis testing and treatment cascades in South Africa: a cross-sectional study. Infect Dis Poverty. (2020) 9:145. 10.1186/s40249-020-00762-8 33092636 PMC7579945

[23] Liu K Zhang Y Qu S Yang W Guo L Zhang L. Prevalence and correlates of anxiety and depressive symptoms in patients with and without multi-drug resistant pulmonary tuberculosis in China. Front Psychiatry. (2021) 12:674891. 10.3389/fpsyt.2021.674891 34557116 PMC8453005

[24] Liu Xianchen TM Hu Lei Wang Aizhen Wu Hongxin Zhao Guifang Gao Chunni. Reliability and validity of the Pittsburgh sleep quality index. Chinese *J Psychiatry*. (1996) 29:103–7. 10.1007/BF02951625

[25] Qiu L Yang Q Tong Y Lu Z Gong Y Yin X. The mediating effects of stigma on depressive symptoms in patients with tuberculosis: a structural equation modeling approach. Front Psychiatry. (2018) 9:618. 10.3389/fpsyt.2018.00618 30534088 PMC6275230

[26] National Health and Family Planning Commission of the People's Republic of China. Tuberculosis classification (WS196-−2017). Elect J Emerg Infect Dis. (2018) 3:191–2. 10.19871/j.cnki.xfcrbzz.2018.03.018

[27] Zhang Y Wang J Lu X Che B Yu J. Sleep status and the associated factors: a large cross-sectional study in Shaanxi Province, China. Int J Environ Res Public Health. (2021) 18:1250. 10.3390/ijerph18031250 33573245 PMC7908339

[28] Koren D Levitt Katz LE Brar PC Gallagher PR Berkowitz RI Brooks LJ. Sleep architecture and glucose and insulin homeostasis in obese adolescents. Diabetes Care. (2011) 34:2442–7. 10.2337/dc11-1093 21933909 PMC3198280

[29] Yew WW Yoshiyama T Leung CC Chan DP. Epidemiological, clinical and mechanistic perspectives of tuberculosis in older people. Respirology. (2018) 23:567–75. 10.1111/resp.13303 29607596

[30] Li J Vitiello MV Gooneratne NS. Sleep in normal aging. Sleep Med Clin. (2018) 13:1–11. 10.1016/j.jsmc.2017.09.001 29412976 PMC5841578

[31] Thomas BE Shanmugam P Malaisamy M Ovung S Suresh C Subbaraman R. Psycho-socio-economic issues challenging multidrug resistant tuberculosis patients: a systematic review. PLoS ONE. (2016) 11:e0147397. 10.1371/journal.pone.0147397 26807933 PMC4726571

[32] Gao W Yang N Mei X Zhu X Hu W Zeng Y. Influence of anti-tuberculosis drugs plus cycloserine on sputum negative conversion rate, adverse reactions and inflammatory factors in multi-drug resistant tuberculosis. Am J Transl Res. (2021) 13:9332–9. 34540050 PMC8430196

[33] Fu L Wang B Chan PSF Luo D Zeng W Ju N. Associations between COVID-19 related stigma and sleep quality among COVID-19 survivors six months after hospital discharge. Sleep Med. (2022) 91:273–81. 10.1016/j.sleep.2021.10.020 34802891 PMC8529895

[34] Kolte IV Pereira L Benites A de Sousa IMC Basta PC. The contribution of stigma to the transmission and treatment of tuberculosis in a hyperendemic indigenous population in Brazil. PLoS ONE. (2020) 15:e0243988. 10.1371/journal.pone.0243988 33326453 PMC7743939

[35] Tsigos C Chrousos GP. Hypothalamic-pituitary-adrenal axis, neuroendocrine factors and stress. J Psychosom Res. (2002) 53:865–71. 10.1016/S0022-3999(02)00429-4 12377295

[36] Buckley TM Schatzberg AF. On the interactions of the hypothalamic-pituitary-adrenal (HPA) axis and sleep: normal HPA axis activity and circadian rhythm, exemplary sleep disorders. J Clin Endocrinol Metab. (2005) 90:3106–14. 10.1210/jc.2004-1056 15728214

[37] Sinha P Lönnroth K Bhargava A Heysell SK Sarkar S Salgame P. Food for thought: addressing undernutrition to end tuberculosis. Lancet Infect Dis. (2021) 21:e318–25. 10.1016/S1473-3099(20)30792-1 33770535 PMC8458477

[38] Okamura K Nagata N Wakamatsu K Yonemoto K Ikegame S Kajiki A. Hypoalbuminemia and lymphocytopenia are predictive risk factors for in-hospital mortality in patients with tuberculosis. Intern Med. (2013) 52:439–44. 10.2169/internalmedicine.52.8158 23411698

[39] Li J Guo L. Association between sleep duration and albumin in US adults: a cross-sectional study of NHANES 2015-2018. BMC Public Health. (2022) 22:1102. 10.1186/s12889-022-13524-y 35655296 PMC9161202

[40] Hui Y Wang X Yu Z Feng H Li C Mao L. Relationship between sleep-wake disturbance and risk of malnutrition in hospitalized patients with cirrhosis. Front Nutr. (2021) 8:719176. 10.3389/fnut.2021.719176 34532336 PMC8439378

[41] Silva FRD Guerreiro RC Andrade HA Stieler E Silva A de Mello MT. Does the compromised sleep and circadian disruption of night and shiftworkers make them highly vulnerable to 2019 coronavirus disease (COVID-19)? Chronobiol Int. (2020) 37:607–17. 10.1080/07420528.2020.1756841 32432519

[42] Pawlowski A Jansson M Sköld M Rottenberg ME Källenius G. Tuberculosis and HIV co-infection. PLoS Pathog. (2012) 8:e1002464. 10.1371/journal.ppat.1002464 22363214 PMC3280977

[43] Deveci F Akbulut HH Celik I Muz MH Ilhan F. Lymphocyte subpopulations in pulmonary tuberculosis patients. Mediators Inflamm. (2006) 2006:89070. 10.1155/MI/2006/89070 16883069 PMC1592589

[44] Care EBoCJoABaCSADoTCBoCIEaPAfMaH. Expert consensus on detection and clinical application of peripheral blood lymphocyte subsets in patients with tuberculosis. Chinese J Antitubercul. (2020) 42:1009–16. 10.3969/j.issn.1000-6621.2020.10.001

[45] Ibarra-Coronado EG Pantaleón-Martínez AM Velazquéz-Moctezuma J Prospéro-García O Méndez-Díaz M Pérez-Tapia M. The bidirectional relationship between sleep and immunity against infections. J Immunol Res. (2015) 2015:678164. 10.1155/2015/678164 26417606 PMC4568388

[46] Irwin M McClintick J Costlow C Fortner M White J Gillin JC. Partial night sleep deprivation reduces natural killer and cellular immune responses in humans. Faseb J. (1996) 10:643–53. 10.1096/fasebj.10.5.8621064 8621064

